# Establishment of CRFK cells for vaccine production by inactivating endogenous retrovirus with TALEN technology

**DOI:** 10.1038/s41598-022-10497-1

**Published:** 2022-04-27

**Authors:** Sayumi Shimode, Tetsushi Sakuma, Takashi Yamamoto, Takayuki Miyazawa

**Affiliations:** 1 Genome Editing Innovation Center, Hiroshima University, Higashi-Hiroshima, Japan; 2 Graduate School of Integrated Sciences for Life, Hiroshima University, Higashi-Hiroshima, Japan; 3 Laboratory of Virus-Host Coevolution, Research Center for Infectious Diseases, Institute for Life and Medical Sciences, Kyoto University, Kyoto, Japan

**Keywords:** Vaccines, Live attenuated vaccines, Microbiology, Virology, Retrovirus, Molecular engineering

## Abstract

Endogenous retroviruses (ERVs) are retroviral sequences present in the host genomes. Although most ERVs are inactivated, some are produced as replication-competent viruses from host cells. We previously reported that several live-attenuated vaccines for companion animals prepared using the Crandell-Rees feline kidney (CRFK) cell line were contaminated with a replication-competent feline ERV termed RD-114 virus. We also found that the infectious RD-114 virus can be generated by recombination between multiple RD-114 virus-related proviruses (RDRSs) in CRFK cells. In this study, we knocked out RDRS *env* genes using the genome-editing tool TAL Effector Nuclease (TALEN) to reduce the risk of contamination by infectious ERVs in vaccine products. As a result, we succeeded in establishing RDRS knockout CRFK cells (RDKO_CRFK cells) that do not produce infectious RD-114 virus. The growth kinetics of feline herpesvirus type 1, calicivirus, and panleukopenia virus in RDKO_CRFK cells differed from those in parental cells, but all of them showed high titers exceeding 10^7^ TCID_50_/mL. Infectious RD-114 virus was undetectable in the viral stocks propagated in RDKO_CRFK cells. This study suggested that RDRS *env* gene-knockout CRFK cells will be useful as a cell line for the manufacture of live-attenuated vaccines or biological substances with no risk of contamination with infectious ERV**.**

## Introduction

Virus-based vaccines are made in living cells. The use of living cells for vaccine manufacturing is a major concern due to the potential presence of oncogenic viruses such as exogenous and endogenous retroviruses in vaccine cell substrates. Inactivating and removing contaminated viruses are essential for vaccine production.

Mammals have hundreds of thousands of copies of retrovirus-related sequences, termed endogenous retroviruses (ERVs), in their genomes. ERVs occupy about 10% of the host genomes of mammals^1^. Although most ERV sequences are disrupted by the accumulation of mutations and deletions, some ERVs retain their open reading frames (ORFs). ERVs retaining ORFs have the potential to produce infectious viral particles when ther are actively transcribed. Measles, mumps, and rubella vaccines (MMR) vaccines and yellow fever vaccines propagated in chicken embryos were reported to be contaminated with endogenous avian leukosis viruses (ALVs) and endogenous avian retroviruses (EAVs)^2,3^. It is unknown whether contaminants are infectious, because these studies only detect the viral RNA, proteins, and reverse transcriptase activities^2,3^. We and others also investigated several live attenuated vaccines for companion animals and revealed that some of them are contaminated with infectious RD-114 virus particles originating from feline ERVs in cell substrates^4–8^. RD-114 virus is a replication-competent feline ERV of ca. 8 kb in length^9^ and several feline cell lines such as Crandell-Rees feline kidney (CRFK) cells produce infectious RD-114 virus^10^. The *env* gene of RD-114 virus has 74% homology with baboon ERV (BaEV), and *gag-pol* genes thought to be derived from feline ERVs. It has been speculated that horizontal transmission from baboons to cats occurred, resulting in the endogenization of the virus in the cat genome^11^. Historically, RD-114 virus was classified as a xenotropic virus^12,13^ (*i.e.*, RD-114 virus cannot infect feline cells); however, we and others reported that RD-114 virus could infect several feline cell lines^14^. We confirmed that feline alanine, serine, cysteine transporter 1 (ASCT1) and 2 (ASCT2) are functional receptors for RD-114 virus infection and they are expressed in various tissues in cats^14–16^. Therefore, RD-114 virus is now considered to be a polytropic virus^14^. ERVs are usually not pathogenic in their original hosts; however, in AKR mice, during aging, an infectious ERV is activated and induces lymphoma^17,18^. Additionally, two independent research groups reported that infectious murine ERVs could exhibit oncogenicity in mice^19,20^. In their studies, replication-competent ERVs were regenerated in immunodeficient mice by recombination between replication-defective ERVs, and the regenerated ERVs induced lymphoma in the hosts. Similar to cases in mice, once the infectious RD-114 virus is regenerated in cats, it may re-infect a wide variety of feline tissues in vivo. The pathogenic potential of the RD-114 virus is unknown at present; however, RD-114 viral expression in domestic cats’ lymphoma and malignant tissues is significantly higher than in normal tissues^21,22^, and a large granular lymphoma cell line, MCC cells, produces infectious RD-114 virus^14^. RD-114 virus also productively infects primary canine epithelial cells as well as canine cell lines^23^. RD-114 viral receptor ASCT2 is also expressed ubiquitously in canine tissues^24^. Therefore, RD-114 virus potentially infects both cats and dogs in vivo.

In the domestic cat genome, there are several copies of RD-114 virus-related sequences (RDRSs) which correspond to a group of ERVs^9,25^. Previously, we identified loci and sequences of six RDRSs^26^. All domestic cats have only one RDRS, located on chromosome C2 and referred to as RDRS C2a, but populations of the other five RDRS s are different depending on the regions where the cats live or breed. Although RDRS C2a has been present in the domestic cat genome for millions of years, its function remains unknown. We demonstrated that infectious RD-114 virus might have been generated by recombination between two RDRSs (RDRS A2 [RDRS on chromosome A2] and RDRS C1 [RDRS on chromosome C1]) in CRFK cells^26^. CRFK cells also have RDRS C2a, but it has stop codons within all viral genes and cannot be the source of infectious RD-114 virus. In this study, to eliminate infectious RD-114 viral particles, we targeted RDRS *env* genes and used the genome-editing tool TAL Effector (TALE) Nuclease (TALEN), which can destroy multiple targets. TALEN consists of DNA-binding domain, TALE, and FokI nuclease domain. The DNA-binding repeat of TALE is composed of tandem 34-amino acid modules, each of which recognizes a single base of DNA^27^. The most widely used genome editing tool, CRISPR-Cas9, is simple to design and use, requiring only 20 bases of target sites to be programmed^28^. However, TALEN remains valuable due to its relatively unconstrained target site and high specificity. The target sequence of CRISPR-Cas9 must be immediately followed by a proto-spacer adjacent motif (PAM) sequence. This target constraint can make it challenging to design and edit targets. Regarding target specificity, CRISPR-Cas9 has been reported to have off-targets that cleave sequences different from the target sequence^29^. TALEN has fewer off-targets because it is a cleavage scheme that induces double-strand breaks only when a pair of TALENs binds correctly to the target sequence, and the target sequence is longer than CRISPR-Cas9. In this study, we chose to use Platinum TALEN, a highly active type of TALEN^30^. Platinum TALEN has been shown to have high cleavage activity in a variety of cells and organisms, including nematodes, sea urchins, newts, frogs, mice, and rats^31^.

## Results and discussion

**Figure 1:**
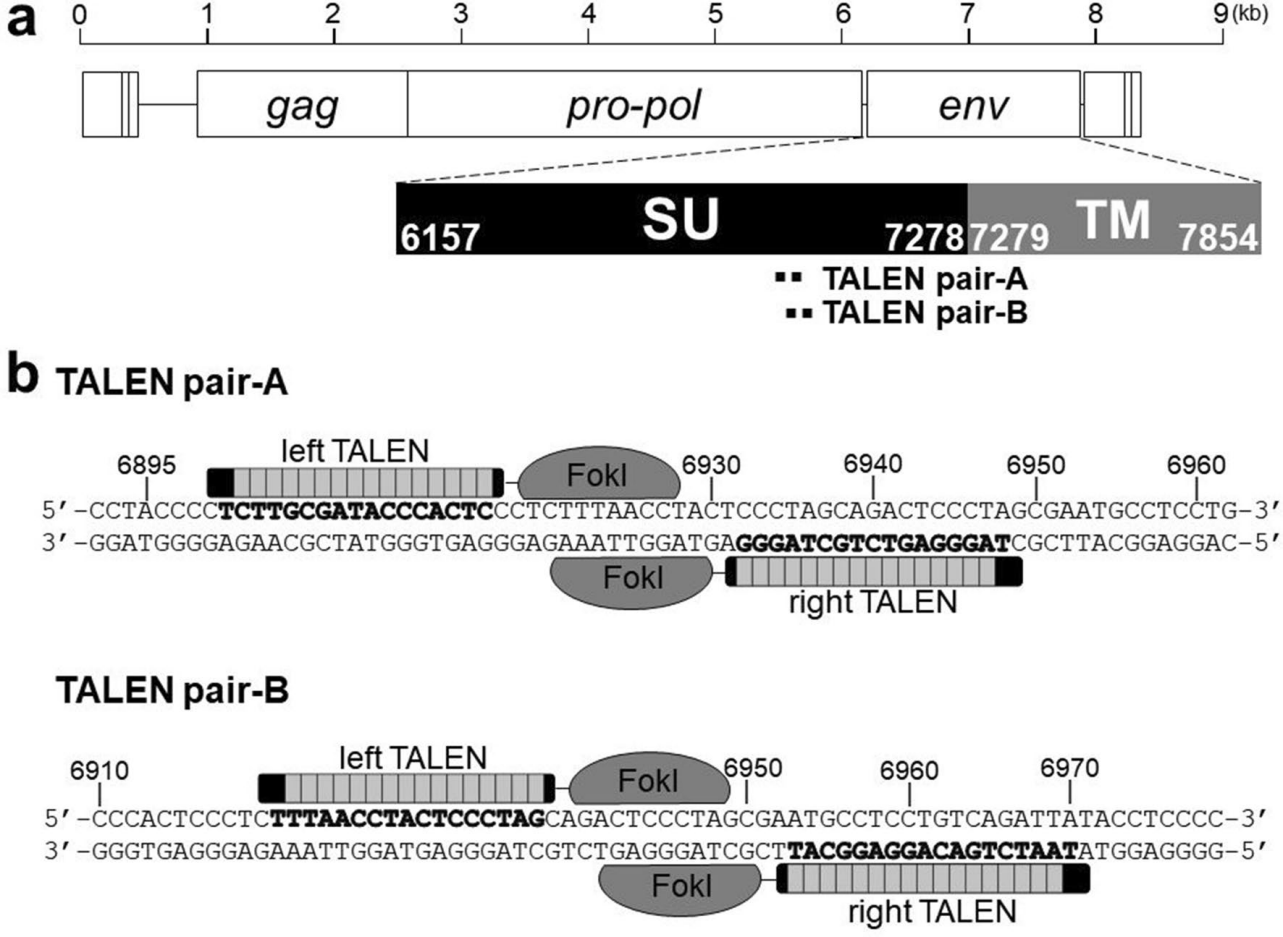
Structures of engineered TALENs binding to RDRS *env* genes. Numbers indicate the nucleotide position in a molecular clone of RD-114 termed pCRT1. (**a**) TALENs target the SU domain of RDRS *env* genes. (**b**) Details of TALEN-targeting sites. Bold characters indicate TALEN target sequences.

**Figure 2:**
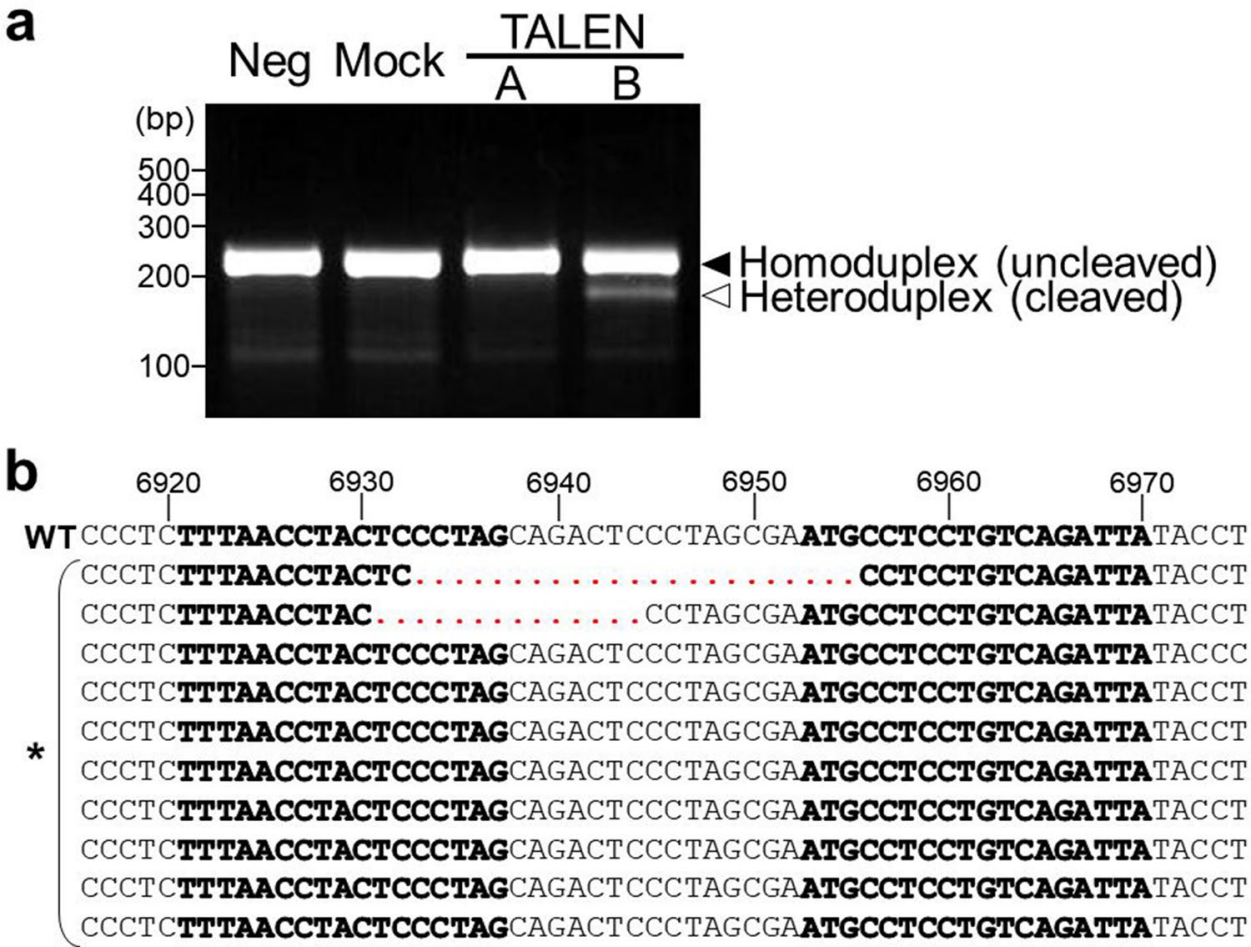
Efficiency of TALEN-induced mutations. (**a**) Surveyor (Cel-I) nuclease assay for TALEN-induced mutations. Solid and open arrowheads indicate the position of the uncleaved PCR fragments (234 bp) and Cel-I cleaved PCR product. Neg, negative control (untreated); Mock, empty vector control (pcDNA3.1); A, TALEN pair-A; B, TALEN pair-B. The gel image has been cropped to focus on the bands of interest by using Microsoft Powerpoint. The original image is presented in Supplementary Fig. S1. (**b**) Sequence analyses of TALEN pair-B-induced mutations. Ten sequences were obtained from genomic DNA of CRFK cells which were transfected with TALEN pair-B plasmid. Numbers indicate the nucleotide position in a molecular clone of RD-114 termed pCRT1. Genomic DNA sequences of TALEN pair-B transfected CRFK cells are shown by an asterisk. Bold characters indicate TALEN pair-B target sequences. Dotted lines in red indicate TALEN pair-B-induced deletions.

**Figure 3:**
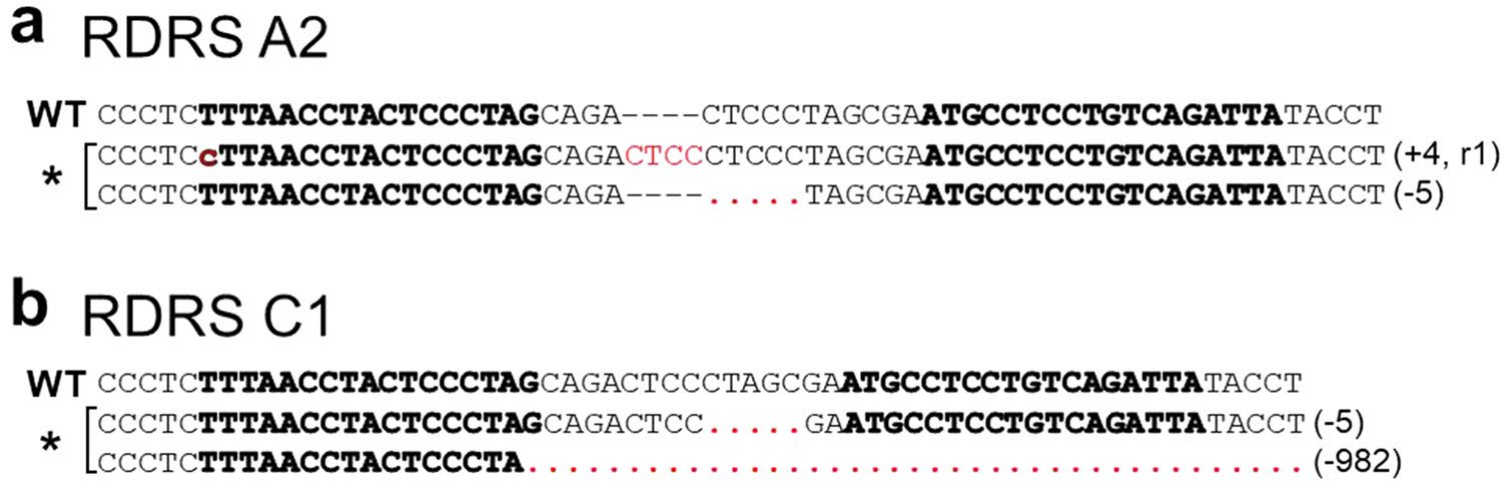
TALEN pair-B-induced mutagenesis of the cloned RDRS *env* KO CRFK cells. Genomic DNA sequences of the TALEN pair-B targeting region of RDRS *env* are indicated. The wild-type (WT) reference sequence (GenBank accession numbers: LC005744 and LC005746) is shown on the top. The target sequences of TALEN pair-B are indicated in bold characters. One out of 31 clones had insertion and deletion mutations on RDRS A2 (**a**) and deletion mutations on RDRS C1 (**b**). Sequences of TALEN-induced mutagenesis of RDKO_CRFK cells are shown by an asterisk. Insertions, deletions and replacement are shown in red by large characters, dotted lines and small characters, respectively. +, inserted nucleotides; −, deleted nucleotides; r, replaced nucleotides.

**Figure 4:**
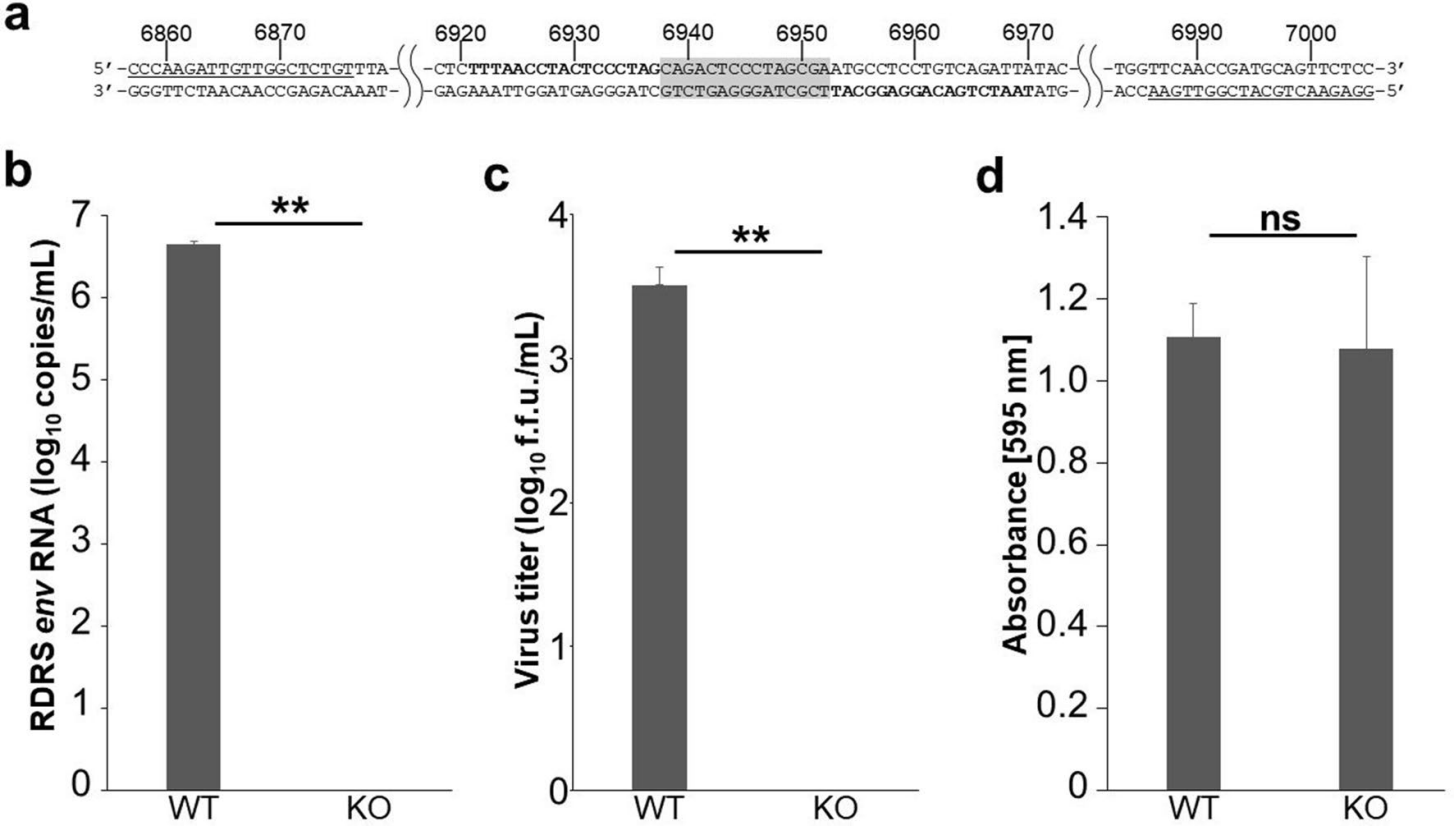
Reduction of RD-114 production in RDRS *env* KO CRFK cells. (**a**,**b**) Parental CRFK (WT) and RDKO_CRFK (KO) cells (1 × 10^6^ cells/well) were cultured for 3 days. The copy numbers of RDRS RNA in the culture supernatant were measured by quantitative real-time RT-PCR using primers and a probe targeting the RDRS *env* region. (**a**) Numbers indicate the nucleotide positions in a molecular clone of RD-114 termed pCRT1. Positions of TALEN pair-B targeting sites are indicated by bold characters. Positions of the TaqMan probe and primers for real-time RT-PCR are shaded in gray, and underlined, respectively. (**b**) Assays were conducted in triplicate for each individual sample. A no-template control was run as a negative control. (**c**) Productions of infectious RD-114 viral particles from parental CRFK (WT) and RDKO_CRFK (KO) cells were quantified by the focus assay. Data represent means ± standard deviation. Significance was assessed by Student’s *t*-test: **p < 0.01. (**d**) Cell viabilities of parental CRFK (WT) and RDKO_CRFK (KO) cells. Parental CRFK and RDKO_CRFK cells were seeded at 1 × 10^5^ cells/well into 96-well plates, and cultured for 2 days. Cell proliferation was analyzed by the MTT assay. The spectrophotometric absorbance of samples was measured at 595 nm. Assays were conducted in triplicate for each sample. Data represent means ± standard deviation. Significance was assessed by Student’s *t*-test: ns, non-significant.

CRFK cells produce infectious RD-114 virus^10^. The infectious RD-114 virus can be regenerated by recombination between two RDRSs (RDRS A2 and C1) in CRFK cells^26^. To prevent the generation of infectious RD-114 virus by recombination, we aimed to knock out *env* genes of RDRS A2 and C1 using TALEN technology, which can destroy multiple targets. Firstly, we designed the TALEN-targeting site on the surface unit (SU) domain of *env* genes (Fig. 1). SU is responsible for binding to a specific receptor on target cells, which is necessary for retroviral infection. We transfected CRFK cells with TALEN plasmids. pcDNA3.1 was used as a negative control. Two days after transfection, genomic DNA was extracted and screened for TALEN-induced mutations using the Surveyor (Cel-I) nuclease assay. Cel-I nuclease recognizes and cuts mismatched heteroduplex DNA formed between wild-type and mutant DNA^32^. TALEN pair-B but not pair-A showed high mutation efficiency (Fig. 2a). Sequencing analysis of RDRS *env* genes revealed that two out of 10 clones contained deletions at the targeted site (Fig. 2b). To generate CRFK cell clones in which *env* genes of both RDRS A2 and C1 were disrupted, we co-transfected CRFK cells with TALEN pair-B plasmid with an expression plasmid of the puromycin-resistant gene. To select cells expressing transfected DNA, a selection marker needs to be co-expressed on either the same construct or on a separate vector that is co-transfected into the cell. In this study, we chose a method to co-transfect with a puromycin-resistant gene plasmid for selection. The TALEN plasmid contains a hygromycin-resistant gene, however, CRFK cells are relatively resistant to hygromycin, making it difficult to select TALEN-transduced cells with hygromycin. Therefore, we cotransfected the puromycin-resistant gene plasmid with TALEN plasmid. CRFK cells are highly sensitive to puromycin, making it easy to select TALEN-transduced cells. Most of the puromycin-resistant clones were also transduced with the TALEN plasmid. One month after puromycin selection, 31 clones were picked up and screened by PCR of genomic DNA and sequencing. In these clones, only one clone was identified as homozygous *env* gene knockout cells on both RDRSs. This clone was re-cloned to ensure clonality, and we named the clone RDKO_CRFK cells. This clone carries different mutations on each allele of the *env* gene of RDRS A2 and C1 (Fig. 3). To confirm whether RDRS *env* disruption results in the inhibition of RD-114 viral production, we cultured parental CRFK and RDKO_CRFK cells for three days and measured the copy numbers of RDRS *env* RNA in the culture supernatants by quantitative real-time RT-PCR (Fig. 4a,b). As a result, we could not detect RDRS *env* RNA in the culture supernatant of RDKO_CRFK cells (Fig. 4b). The S+L− focus assay indicated that the infectious titer of RD-114 virus produced from RDKO_CRFK cells was below the detection limit (Fig. 4c). We have passaged RDKO_CRFK cells four times to investigate if they produced RD-114 virus. When a long-term culture is performed, the defective RDRSs may cause recombination again if it transcribed in RDKO_CRFK cells. However, we have confirmed that there are only three copies of *env* genes in the CRFK cell genome in previous report^26^ and that all of them have been disrupted; *env* gene of RDRS C2a also disrupted in RDKO_CRFK cells (Supplementary Fig. S2). Therefore, there is no possibility of producing RD-114 virus with a full-length *env* gene. The cell viability of RDKO_CRFK cells was similar to that of parental CRFK cells, indicating that the *env* knockout does not affect the proliferation of CRFK cells (Fig. 4d).

**Figure 5:**
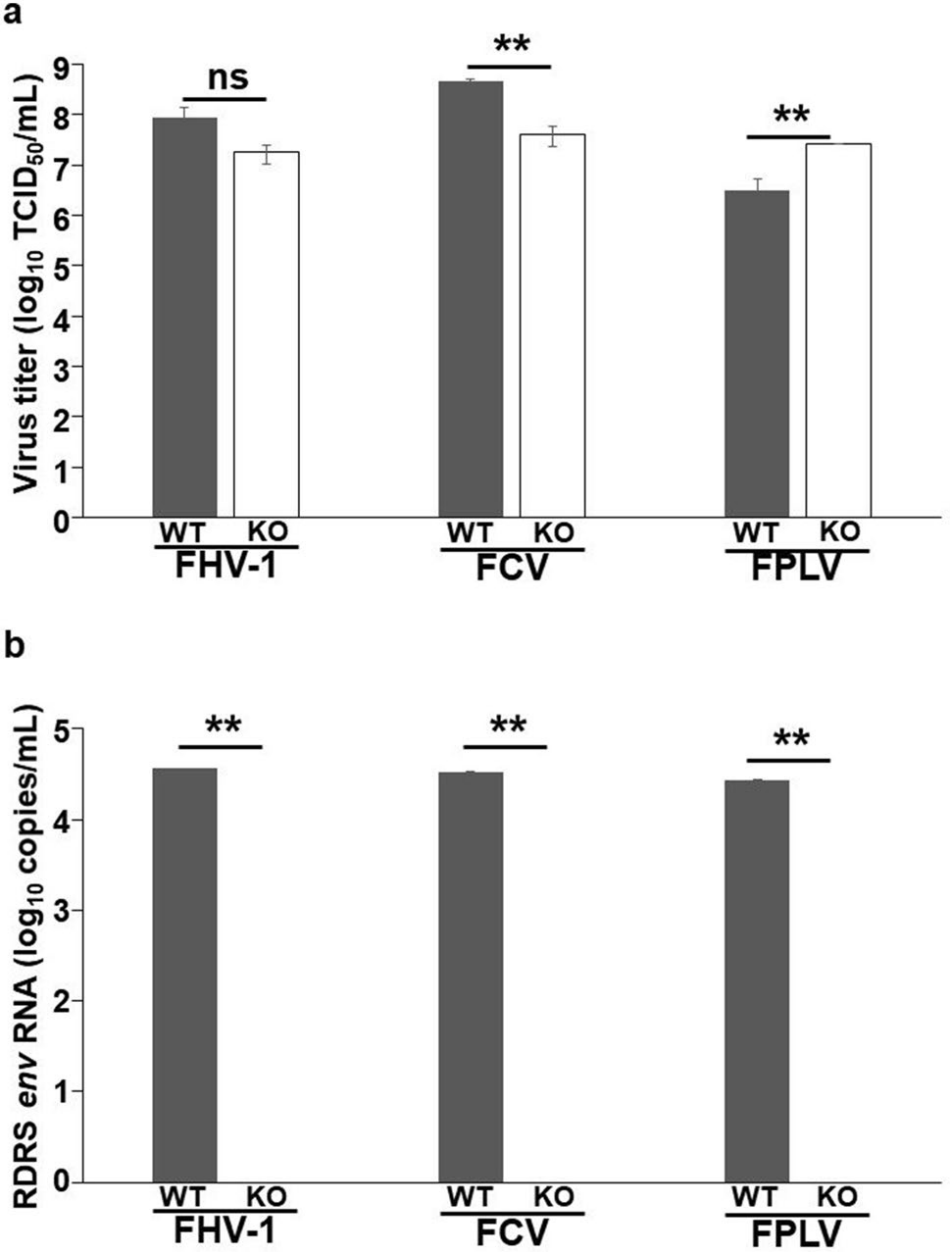
Propagation of feline panleukopenia virus, calicivirus, and herpesvirus in RD-114 virus KO CRFK cells. Parental CRFK (WT) and RDKO_CRFK (KO) cells were inoculated with FHV-1 strain 00-015 at 1000-fold dilutions of a virus stock, FCV strain 01-106 at a multiplicity of infection (MOI) of 0.01, or FPLV strain V142 at MOI of 0.1. (**a**) The titers of FVRV and FCV produced in parental CRFK or RDKO_CRFK cells were measured in TCID_50_ using CRFK cells. The titers of FPLV produced in parental CRFK or RDKO_CRFK cells were measured in TCID_50_ using FL74 cells. Assays were conducted in triplicate for each individual sample. Data represent means ± standard deviation. Significance was assessed by Student’s *t*-test: **p < 0.01; ns, non-significant. (**b**) RDRS *env* RNA in the viral stocks propagated in parental CRFK or RDKO_CRFK cells were quantified by real-time RT-PCR, as done in Fig. 4. Data represent means ± standard deviation. Significance was assessed by Student’s *t*-test: **p < 0.01.

CRFK cells have been used to propagate several viruses for vaccines for animals, including core feline trivalent inactivated vaccines called “FVRCP vaccines” against feline herpesvirus type 1 (FHV-1), calicivirus (FCV), and panleukopenia virus (FPLV). We examined the effects of *env* knockout on the growth of FHV-1, FCV, and FPLV. Parental CRFK and RDKO_CRFK cells were inoculated with FHV-1, FCV, and FPLV and cultured for 4, 2, and 6 days, respectively, and the titer (expressed as TCID_50_) of each virus was measured. The titer of FHV-1 in RDKO_CRFK cells was similar in the parental cells, but the titer of FCV was significantly lower and that of FPLV was significantly higher (Fig. 5a). These results indicate that the expression of RDRS had some effect on FHV-1, FCV and FPLV growth. Further analyses, for this reason, would be required. We also measured the copy number of RDRS *env* RNA in the viruses produced in both parental CRFK and RDKO_CRFK cells by real-time RT-PCR. The copy numbers of RDRS *env* RNA in the preparation of FHV-1, FCV, and FPLV from RDKO_CRFK cells were below detection limits (Fig. 5b).

Finally, to investigate the potential off-target effects of TALEN, we first searched the reference sequence Felis_catus_8.0 (GenBank accession number: GCA_0001811335.3) for sites that shared similar sequences with TALEN pair-B target. The top ten potential off-target sites were sequenced. None of the sequences of off-target candidates were mutated (Table 1).

**Table 1:** Potential off-target sites of RDRS *env.*

Name	Sequence	TALEN score (average)	Accession number	Off target gene name
On-target	5′-*TTAACCTACTCCCTAG*CAGACTCC	5.16	LC005744–LC005746	–
	CTAGCGA**ATGCCTCCTGTCAGATTA**-3′			
Off-target1	5′-**TAATGAAACAGCTGGCAA**GCTTTATCT	14.87	NT_212387.1	–
	TCTCGTTT**ATGCTTTCTGGCAGGTTA**-3′			
Off-target2	5′-**TAAACAAACTAGAAGCAT**TCCATTGATGGTAA	15.035	NT_233441.1	–
	ACCTGATGCCGTTT**AGGCTTCTTTTTTGTTTA**-3′			
Off-target3	5′-**TAACCCAAAAGAAAGCAA**GAAAGCAGATA	15.81	NT_211338.1	PPP1R9A
	AAAATAACAAG**GATGTTTTTGTTAGATTA**-3′			
Off-target4	5′-**TAAAAAAACAAAAAACAA**ACAAACAACAACA	15.83	NT_191194.1	GPC6
	ACAAACTCTACT**ATTCTTAGTGTTAGATCA**-3′			
Off-target5	5′-**TAATCTGACAAATTACAT**TAATTAGA	15.915	NT_235977.1	–
	TATGTATT**ATCTTACATGTTAGCTTA**-3′			
Off-target6	5′-**TCATCTCACCAAAGCCAT**ATCAGTAT	15.96	NT_223984.1	SPRED2
	TTAAAAAT**ATTGTTCCTATCTGATAA**-3′			
Off-target7	5′-**TAATGTATCAGAAGATAT**GCAAA	16.28	NT_196674.1	–
	TAGCT**ATTTCTTTTGTTAGAAGA**-3′			
Off-target8	5′-**TCATCTAACATAACTCAG**CTTCCTTTT	16.565	NT_225093.1	GRID2
	CAACCCTTT**ATTCTTTCTTTTTTATTA**-3′			
Off-target9	5′-**TCCTCTTACAGCCCAAAT**AAAATTG	16.685	NT_244747.1	–
	GACTTTT**TTGGCTCCTGTTTTATTA**-3′			
Off-target10	5′-**TAATCTGACCACAGCATT**TGAAAAAGT	16.69	NT_192414.1	PDE4D
	AGTGTTTT**ATTCTTGCTTTGAAATTA**-3′			

Italics and bold characters indicate left and right TALEN target sequences, respectively. Mismatches are underlined.

There are several cases of ERV-contaminated vaccines for not only companion animals but also humans. MMR vaccines and yellow fever vaccines grown in chicken embryo fibroblasts were contaminated with endogenous ALVs and endogenous EAVs, originating from chicken embryonic fibroblast substrates^2,3^. Herpes virus 3 vaccine grown in human embryonic lung cells was contaminated with human ERV K^33^. Our results may be useful for the manufacture of live-attenuated vaccines with no risk of contamination with infectious ERVs. Recently, it was reported that all 62 copies of the porcine endogenous retroviruses (PERVs) were disrupted in a pig kidney epithelial cell line by CRISPR-Cas9 mediated genome editing^34^ and generated PERV-inactivated pigs via somatic cell nuclear transfer^35^. Our strategy to control ERV production as well as the method of PERV knockout, can be used to treat other cell lines for vaccine production, but also for studies of the function of ERVs.

## Methods

### Cell cultures

CRFK (CCL-94, ATCC, Manassas, VA, USA), AH927 (feline embryonic fibroblasts)^36^, and a feline sarcoma-positive leukemia-negative (S+L−) fibroblast cell line termed QN10S cells^37^ were cultured in Dulbecco's modified Eagle's medium (DMEM) (Sigma-Aldrich St. Louis, MO, USA) supplemented with 10% heat-inactivated fetal calf serum (FCS) (Equine-biotech, Bengaluru, India), penicillin (100 IU/mL), and streptomycin (100 ng/mL) (Invitrogen, MA, USA ). FL74 cells (feline lymphoblasts) (ATCC CRL-8012)^38^ were cultured in RPMI 1640 medium (Sigma-Aldrich) supplemented with 10% heat-inactivated FCS, penicillin (100 IU/mL), and streptomycin (100 ng/mL).

### Construction of TALEN plasmids

A two-step Golden Gate assembly method with the Platinum Gate TALEN Kit (Addgene, MA, USA)^30^ was used to construct Platinum TALEN plasmids containing the homodimer-type *Fok*I nuclease domain. TALENs were designed against the RDRS *env* gene. Target sequences for TALENs are summarized in Fig. 1.

### Knockout of RDRS *env* in CRFK cells

CRFK cells (5 × 10^5^) were suspended in 100 µL of R buffer (Neon Transfection system, Invitrogen) and then electroporated with 1.8 µg of each TALEN plasmid and 0.36 µg of a puromycin-resistant gene expression plasmid to select TALEN-transfected cells under the following conditions: pulse voltage, 1400 V; pulse width, 20 ms; and pulse number, 2. This transfection procedure was repeated twice.

### Cel-I assay and sequencing

Two days after transfection, genomic DNA was extracted from CRFK cells using QIAamp DNA Blood Mini kit (QIAGEN, CA, USA). PCR was performed using PrimeSTAR GXL polymerase (TaKaRa, Shiga, Japan) according to the manufacturer’s instructions. Primers used for this PCR were listed in Table 2. The amplicons were heat-denatured, digested by Surveyor nuclease (Transgenomic, NE, USA), and subjected to agarose gel electrophoresis to detect TALEN-induced mutations.

**Table 2:** Primers and probe used in this study.

Assay	Target	Sequence	Position	Reference sequence
Cel-I assay	RDRS *env*	5′-CCCTCTTGCGATACCCAC-3′	6897–6914	AB559882
		5′-GACCCGTTTAGGGCACATAA-3′	7111–7130	
Screening	RDRS A2	5′-CCCAACAGGAATGGTCATTTTATG-3′	6180–6203	LC005744
		5′-CTGTAACAGACTTTCATAAGAG-3′	A2:152395721–152395742	Felis_catus_8.0
	RDRS C1	5′-CCCAACAGGAATGGTCATTTTATG-3′	6234–6257	LC005746
		5′-GAGCAGTTACAGCATTTACCC-3′	C1: 60469429–60469449	Felis_catus_8.0
Real-time RT-PCR	RD-114 *env*	5′-CCCAAGATTGTTGGCTCTGT-3′	6857–6876	AB559882
		5′-GGAGAACTGCATCGGTTGAA-3′	6986–7005	
		5′-FAM-CAGACTCCCTAGCGA-MGB-3′	6938–6952	
Off-target	Off-target1	5′-GAAGAAGAACAAAAGCTGGGG-3′	124–144	NT_212387.1
		5′-ACATAACCCCCAGCACAGAA-3′	262–281	
	Off-target2	5′-CCGCTCACACTCTATCTCTCAA-3′	370–391	NT_233441.1
		5′-ATAAGATATCAAATGTGCCAGAAAT-3′	590–614	
	Off-target3	5′-TACTGGTTATAAGGGAGAGT-3′	201–220	NT_211338.1
		5′-TTTGTAATCTAACAAAAACATC-3′	493–514	
	Off-target4	5′-GCCATCAGTATGAGACAGACAA-3′	48–69	NT_191194.1
		5′-ATTCCAGGTCTGTGCTTGCT-3′	243–262	
	Off-target5	5′-TAAGTTAGCTGCAACGGGCA-3′	78–97	NT_235977.1
		5′-TCAGGGCCAGTATATCCTCA-3′	352–371	
	Off-target6	5′-CCAAAATCTACTCCCAATACCAG-3′	101–123	NT_223984.1
		5′-TGCCAAGTGACTGGTTAAAAG-3′	281–301	
	Off-target7	5′-CCCTTTTTGTGGACTTCAGG-3′	252–271	NT_196674.1
		5′-CAAAGCTCACTTTTCCCCAC-3′	522–541	
	Off-target8	5′-TGGAGCATCTACTTGGCTCA-3′	7680–7699	NT_225093.1
		5′-AAGGGAGGAGGGAACAAAGA-3′	7864–7883	
	Off-target9	5′-AACAACGGCAAGAGAGAGGA-3′	24–43	NT_244747.1
		5′-TTTCAGTCGGGAGCTATGGT-3′	274–293	
	Off-target10	5′-GCATCTGCCATGTGTGTGTA-3′	242–261	NT_192414.1
		5′-TAGTGGTGACTTTGGCCTGA-3′	449–468	

The PCR products were also cloned into pCR BluntII TOPO plasmid vector (Life Technologies, CA, USA) and sequenced. Sequencing was performed by a commercial DNA sequencing service (FASMAC, Kanagawa, Japan). Alignment of nucleotide sequences and estimation of homology were performed using GENETYX win Ver.11 (GENETYX, Tokyo, Japan).

### Selection of TALEN-transfected CRFK cells

TALEN-transfected CRFK cells were seeded in 10-cm dishes and selected with 4 μg/mL of puromycin (InvivoGen, CA, USA). Then, AH927 cells, which do not contain RDRS A2 or C1 in their genomes (thus, AH927 cells are RD-114 non-producer cells), were co-cultured as feeder cells to supply the nutrients for the small colonies. Medium with puromycin was replaced every 3 days. One month after starting selection with puromycin, individual puromycin-resistant cell colonies were picked up and cultured in 96-multiwell plates. Two days after transferring the cells into 96-multiwell plates, the cells were further subcultured in 6-multiwell plates. The clones were then subjected to PCR and sequencing analyses using genomic DNA.

### Quantification of RD-114 viral production by real-time RT-PCR

The untreated parental CRFK cells and cloned RDRS *env* knockout CRFK cells (RDKO_CRFK cells) were cultured for 3 days. Culture supernatants were collected, filtrated through 0.45-µm filters, and then pretreated with DNase I (Roche Diagnostics, IN, USA). The copy number of RDRS *env* RNA in the culture supernatants was measured by real-time RT-PCR. Real-time RT-PCR was performed using TaKaRa Ex Taq HS DNA polymerase (TaKaRa) according to the manufacturer’s instructions. A TaqMan probe (Applied Biosystems, MA, USA) and primers used for real-time RT-PCR were listed in Table 2.

### Titration of infectious RD-114 virus

Replication-competent gammaretrovirus in the culture supernatants of parental CRFK and RDKO_CRFK cells was evaluated by the S+L− assay using QN10S cells^39^. QN10S cells were seeded in 24-well plates at 1 × 10^4^ cells/well one day before infection and diluted supernatants of RDKO_CRFK cells were used to inoculate QN10S cells in the presence of polybrene (Sigma-Aldrich) (8 µg/mL). Seven days after inoculation, numbers of foci were counted.

### Cell proliferation assay

Cell proliferations of parental CRFK and RDKO_CRFK cells were measured using Cell Proliferation Kit I (MTT) (Roche Diagnostics). The cells were seeded at 1 × 10^5^ cells/well in 96-multiwell plates and then cultured for 2 days. The assay was performed according to the manufacturer’s instructions. The spectrophotometric absorbance of the sample was measured at a wavelength of 595 nm using a Wallac 1420 ARVOsx (PerkinElmer Life Sciences, MA, USA).

### Infection and titration of feline herpesvirus type 1, calicivirus, and panleukopenia virus

FHV-1 strain 00-015^40^, FCV strain 01-106, and FPLV strain V142^41^ were used. To evaluate the growth kinetics of FHV-1 strain 00-015, 1000-fold dilutions of a virus stock are prepared, and 60 µL aliquots are used to inoculate parental CRFK and RDKO_CRFK cell monolayers in 6-multiwell plates. The cells were inoculated with FCV strain 01-106 at a multiplicity of infection (MOI) of 0.01 and FPLV strain V142 at MOI of 0.1. After incubation at 37 °C for 1 h, the cells were washed three times with PBS and then cultured with 2 mL of DMEM supplemented with 10% FCS for 4, 2, and 6 days, respectively. The titers of FHV-1 and FCV produced in parental CRFK or RDKO_CRFK cells were measured in TCID_50_ using CRFK cells, as described previously^40,42^. The titers of FPLV produced in parental CRFK or RDKO_CRFK cells were measured in TCID_50_ using FL74 cells, as described previously^43,44^.

### Off-target analysis

The Paired Target Finder (https://tale-nt.cac.cornell.edu/)^45^ was used to identify potential off-target sites for RDRS *env*. Score cutoff and spacer lengths were set to 3.0 and 10–30, respectively. Genomic regions around each candidate site were amplified by PCR using primers listed in Table 2 and the sequences were confirmed by direct sequencing.

## Supplementary Information

Supplementary Figures.

## Data Availability

All data presented in this study are available from the corresponding author upon reasonable request.
