# Supplementary material for: Establishment of CRFK cells for vaccine production by inactivating endogenous retrovirus with TALEN technology
Source: Sci Rep. 2022 Apr 27;12:6641. doi: 10.1038/s41598-022-10497-1 (PMC9046391; doi:10.1038/s41598-022-10497-1)
Supplement: Supplementary file 1 — Supplementary Figures. [file 41598_2022_10497_MOESM1_ESM.docx]

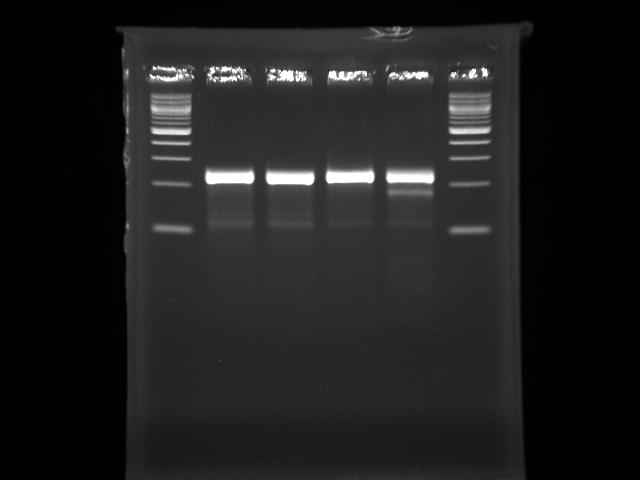


**Supplementary Figure 1. Full-length agarose gel of Surveyor (Cel-I) nuclease assay for TALEN-induced mutations.**

Uncropped and unprocessed version of Fig. 2 in the main text. The lanes on both ends indicate 100 bp DNA ladder (NEB, Ipswich, MA, USA).


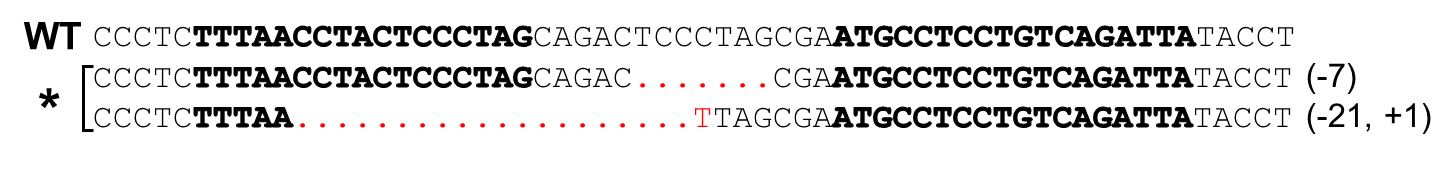


**Supplementary Figure 2. TALEN pair-B-induced mutagenesis of the cloned RDRS *env* KO CRFK cells.**

Genomic DNA sequences of the TALEN pair-B targeting region of RDRS C2a *env* are indicated. The wild-type (WT) reference sequence (GenBank accession numbers: LC005745) is shown on the top. The target sequences of TALEN pair-B are indicated in bold characters. One out of 31 clones had insertion and deletion mutations on RDRS C2a. Sequences of TALEN-induced mutagenesis of RDKO_CRFK cells are shown by an asterisk. Insertions and deletions are shown in red by large characters and dotted lines, respectively. +, inserted nucleotides; -, deleted nucleotides.
